# Glacial History and Landscape Features Shape the Hierarchical Population Genetic Structure of Woodland Caribou (*Rangifer tarandus caribou*) in Western Canada

**DOI:** 10.1111/eva.70269

**Published:** 2026-05-22

**Authors:** Samuel Deakin, Anita Michalak, Maria Cavedon, Charlotte Bourbon, Margaret M. Hughes, Lalenia Neufeld, Agnès Pelletier, Jean Polfus, Helen Schwantje, Robin Steenweg, Caeley Thacker, Madeline Trottier, Marco Musiani, Jocelyn Poissant

**Affiliations:** ^1^ Faculty of Veterinary Medicine University of Calgary Calgary Alberta Canada; ^2^ Department of Biological Sciences University of Calgary Calgary Alberta Canada; ^3^ Jasper National Park of Canada Parks Canada Jasper Alberta Canada; ^4^ British Columbia Ministry of Water, Land, and Resource Stewardship Terrestrial Species Recovery Branch Prince George British Columbia Canada; ^5^ Canadian Wildlife Service – Pacific Region Environment and Climate Change Canada Kelowna British Columbia Canada; ^6^ Department of Biology University of British Columbia Kelowna British Columbia Canada; ^7^ (Emeritus) Wildlife and Habitat Branch Ministry of Water, Lands, and Natural Resource Stewardship, Government of British Columbia Nanaimo British Columbia Canada; ^8^ Wildlife and Habitat Branch Ministry of Water, Lands, and Natural Resource Stewardship, Government of British Columbia Nanaimo British Columbia Canada; ^9^ Dipartimento Scienze Biologiche Geologiche Ambientali Università di Bologna Bologna Italy

**Keywords:** conservation genomics, endangered species, evolutionarily significant units, genetic differentiation, glacial history, population structure

## Abstract

Characterising hierarchical population structure is crucial to understanding a species' evolutionary history and informing effective conservation and management strategies. Many terrestrial species in North America have experienced a wide range of evolutionary pressures at multiple scales, ranging from large‐scale range shifts and recolonisations driven by glacial cycles to more localized contemporary habitat degradation and fragmentation. Hence in this region, given the multi‐level evolutionary forces at play, genetic variation and diversity are often hierarchically structured. We analysed genomic diversity and variation in woodland caribou ( *Rangifer tarandus caribou* ) across western Canada using genotypes from ~33,000 Single Nucleotide Polymorphism (SNP) loci from 759 geo‐referenced individuals spanning 45 pre‐defined subpopulations. We employed genetic clustering methods and measures of genetic differentiation to characterise hierarchical population structure in the region and tested for latitudinal changes in heterozygosity resulting from post‐glacial recolonisation and hybridisation. Our results confirm that woodland caribou genetic diversity and differentiation occur at multiple hierarchical levels, reflecting post‐glacial recolonisation patterns and landscape heterogeneity. Notably, the major genetic clusters identified in our study do not align with current recognised units for the species in this region. We also observe elevated heterozygosity in the mid‐latitudes of the sampled range, indicative of hybridisation following secondary contact during post‐glacial recolonisation. These findings underscore the need to consider and include genetic diversity at all hierarchical levels in conservation planning, as wide‐ranging species often experience diverse and complex evolutionary histories and pressures.

## 1 Introduction

The population genetic structure of a species provides valuable insights into both its historical and contemporary dynamics (Hewitt 2004; Shafer et al. 2010). Patterns of genetic differentiation such as variation in diversity and connectivity across a species' range can reveal past colonisation and recolonisation events (Deakin et al. 2020; Shafer et al. 2011; Stone and Cook 2000). Additionally, these patterns inform our understanding of contemporary gene flow, shedding light on the biotic and abiotic factors that shape genetic connectivity (Breistein et al. 2022; Epps et al. 2018; Forbes and Hogg 1999). Understanding the natural history and contemporary gene flow of a species can be crucial for its management and conservation, particularly in defining and prioritising conservation units below the species level (Crandall et al. 2000; Fraser and Bernatchez 2001; Hoelzel 2023; Moritz 1994).

Often, the processes that cause population structure do not act in isolation and can occur at different temporal and spatial scales, leading to a phenomenon known as hierarchical population structure (Vähä et al. 2007). For instance, some anadromous fish are structured by both major rivers and sub‐structure within water catchments (Poissant et al. 2005; Vähä et al. 2007). Similarly, terrestrial species often show major discontinuities in population structure due to past glacial refugia (Shafer et al. 2010), and finer substructure due to localized geographic features such as rivers, mountains and differing habitat types (Cross et al. 2016; Deakin et al. 2020; Jenkins et al. 2018; Sim et al. 2019). Identifying nested population subdivisions can be challenging for species with broad distributions due to the complex interactions of historical and ecological processes, as well as the need for conducting adequate sampling across vast geographic areas (Cheeseman et al. 2019; Warnock et al. 2010). However, recognizing hierarchical structure is essential for describing population structure, as it helps identify and delineate evolutionary significant lineages which may be obscured when only considering local or broad‐scale differentiation (Cross et al. 2016; Warnock et al. 2010).

In the conservation of species, the characterisation of unique genetic, ecological, or behavioural adaptation below the species level can help identify populations upon which to focus conservation efforts (Crandall et al. 2000; Fraser and Bernatchez 2001; Hoelzel 2023; Moritz 1994; Ryder 1986). In the United States, conservation units are often identified, managed and protected as Evolutionarily Significant Units, which from a genetic standpoint are used to identify, delineate and preserve groups of animals or plants containing unique and putatively‐adaptive genetic variation (Crandall et al. 2000; Fraser and Bernatchez 2001; Hoelzel 2023; Moritz 1994). Similarly, in Canada, conservation units are identified and managed as Designatable Units (DUs) listed under the Species at Risk Act (SARA). These DUs are intended to capture unique, significant and irreplaceable components of Canada's biodiversity (COSEWIC 2011; Harding 2022; Muir et al. 2021).

Caribou ( *Rangifer tarandus* ) and their conspecifics, reindeer, have a circumpolar distribution (Flagstad and Røed 2003; Yannic et al. 2014) and inhabit diverse regions and habitat types (Harding 2022). Like many other terrestrial species, caribou recolonised northwestern North America following the last glacial maximum from at least two refugia; the Beringian refugium, located in northeastern Siberia and northwestern North America and the North American refugium, consisting of much of the present‐day mainland United States (Shafer et al. 2010; Hewitt 2004; Flagstad and Røed 2003; Yannic et al. 2014). Woodland caribou (*R. t. caribou*, Banfield 1961) inhabit the southern regions of the species' distribution in North America and stem from two lineages reflective of their glacial history; the Beringian‐Eurasian Lineage (BEL) and the North American Lineage (NAL) (Cavedon, Poissant, et al. 2022; McDevitt et al. 2009; Polfus et al. 2017; Yannic et al. 2014). Within woodland caribou, various ecotypes and subpopulations are observed (Theoret et al. 2022), which exhibit behaviours and adaptations linked to their ancestral lineages (Cavedon, vonHoldt, et al. 2022; McDevitt et al. 2009). As previously noted by Michalak (2023), given their broad distribution, presence of differing glacial ancestries and previously identified population structure (McLoughlin et al. 2004; Priadka et al. 2019; Serrouya et al. 2012; Wilson et al. 2022), woodland caribou in western Canada likely exhibit a hierarchical population structure.

**FIGURE 1:**
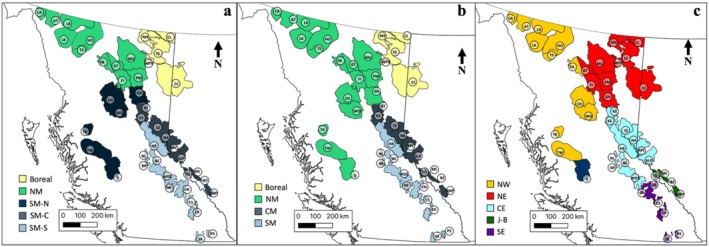
Distribution of studied woodland caribou subpopulations in western Canada, with labels of genotyped subpopulations following the abbreviation scheme in Table 1. (a) Depicts classification of subpopulations according to the Species at Risk Act (SARA) Schedule 1 populations: Boreal population, Northern Mountain (NM) population and Southern Mountain population (SM) Northern (‐N), Central (‐C) and Southern (‐S) groups. (SARA 2014) (b) Depicts Designatable Units (DUs) (COSEWIC 2011) classification proposed in 2014 by the Committee on the Status of Endangered Wildlife in Canada: Boreal population (DU6), Northern Mountain population (NM; DU7), Central Mountain population (CM; DU8) and Southern Mountain population (SM; DU9). (c) Depicts major genetic clusters inferred from our analyses. Here, the Frog population is classified as a northwestern population as it is situated west of the Rocky Mountain Trench despite its minor‐majority assignment to the northeastern cluster (an overall assignment rate of 50.3%). Given that this subpopulation contained near equal portions of northeastern and northwestern genetic backgrounds, the Frog subpopulation likely represents a transition zone between the two northern clusters. Maps created in QGIS.

In Canada, caribou subpopulations, often referred to as ‘herds’, are grouped into DUs or SARA‐listed units for conservation purposes (Weckworth et al. 2018). In western Canada, the focal region of this study, caribou DUs and SARA delineations are in part attributed to both phylogeographic history and population genetic structure (Cronin et al. 2005; Klütsch et al. 2016; McDevitt et al. 2009; Polfus et al. 2017; Serrouya et al. 2012; Taylor et al. 2021; Weckworth et al. 2012; Yannic et al. 2014); however, many previous studies have used different sets of genetic markers and examined different subpopulations, making it difficult to compare results. At present, four DUs and three SARA‐listed units (which overlap the DUs) have been identified in this region (COSEWIC 2011; SARA 2012a, 2012b, 2014). In both classification schemes, boreal, northern mountain and southern mountain populations are differentiated, with additional partitioning within the southern mountain population (Figure 1).

We build on the preliminary work of Michalak (2023) to conduct a comprehensive hierarchical analysis of genetic diversity and variation in woodland caribou across western Canada. Specific objectives were to: (i) identify broad‐ and fine‐scale population genetic structure and its causes, and (ii) test if genetic diversity was higher in the central region of our sampling range due to secondary contact and hybridisation between glacial lineages. To do this we used genotypes from ~33,000 loci generated using a caribou‐specific Single Nucleotide Polymorphism (SNP) array. Given the expectation of hierarchical population structure and the limited resolution of previous genetic studies of caribou in western Canada, we hypothesised that genetic structure may not fully align with current management schemes. Specifically, we expected to find two overarching clusters representing differing glacial ancestries, below which we expected landscape features to explain further sub‐structure. As for genetic diversity, we expected it would be highest in the middle of the studied range, where the two glacial lineages of caribou are known to have hybridised. This study provides insights into the natural history, contemporary gene flow and factors affecting the population genetic structure of caribou in western Canada.

## 2 Materials and Methods

### 2.1 Samples and Geographic Data

Woodland caribou blood and tissue samples were collected by Provincial Government and Parks Canada partners in British Columbia and Alberta, Canada, between 2012 and 2023. Due to the sampling regimes of these partners, most samples came from female animals. All samples were associated with a georeferenced sampling location and/or range of origin.

### 2.2 DNA Extraction and Genotyping

DNA was prepared following the protocols described in Michalak (2023). In brief, DNA was extracted using a QIAGEN DNeasy Blood & Tissue or QIAamp 96 DNA QIAcube HT Kit with recommended manufacturer protocols and eluted in 400 μL of molecular grade water. DNA was then quantified using either a BioTek Synergy LX Multimode Reader or Thermo Fisher Qubit 4 Fluorometer and the Thermo Fisher Quant‐iT and Qubit dsDNA Assay Kits, respectively. Eight hundred and fifty‐four samples from 45 pre‐defined subpopulations (Table 1, Figure 1) containing ≥ 400 ng of DNA were normalized to a quantity of 400 ng, dried on a Thermo Scientific Savant SpeedVac DNA 130 Integrated Vacuum Concentrator System and sent to the Centre d'expertise et de service Génome Québec (Montreal, Canada) for genotyping using an Illumina single nucleotide polymorphisms (SNP) array targeting ~60,000 loci distributed across the caribou genome (Carrier et al. 2022).

**TABLE 1:** Genetic variation across woodland caribou subpopulations in western Canada.

Subpopulation	Abbr	Pop est	Total genotypes	Non relatives	*H*_*o*_ ± s.d.	*H*_*e*_ ± s.d.	*F*_IS_	DU	SARA	Structure level 1	Structure level 2	Structure level 3
Itcha‐Ilgachuz	II	559	77	75	0.33 ± 0.17	0.33 ± 0.17	0.00	DU7	SM‐N	II	II	II
Atlin	AT	1527	19	19	0.38 ± 0.17	0.37 ± 0.14	−0.03		NM	All other subpops	Northern cluster	NW
Carcross	CA	851	6	5	0.37 ± 0.24	0.33 ± 0.16	−0.12					
Horseranch	HO	600	4	4	0.39 ± 0.26	—	—					
Level Kawdy	LK	1500	3	3	0.39 ± 0.30	—	—					
Little Rancheria	LR	752	7	7	0.39 ± 0.22	0.36 ± 0.14	−0.08					
Tsenaglode	TS	840	7	7	0.39 ± 0.21	0.36 ± 0.14	−0.08					
Chase	CH	600	45	40	0.39 ± 0.13	0.39 ± 0.12	0.00		SM‐N			
Telkwa	TK	31	5	5	0.38 ± 0.25	0.33 ± 0.17	−0.15					
Tweedsmuir	TW	178	29	28	0.35 ± 0.17	0.34 ± 0.16	−0.03					
Wolverine	WO	330	42	38	0.38 ± 0.14	0.39 ± 0.12	0.03					
Graham	GH	128	22	22	0.40 ± 0.15	0.39 ± 0.12	−0.03					NE
Calendar	CL	231	9	9	0.40 ± 0.20	0.37 ± 0.13	−0.08	DU6	Boreal			
Chinchaga	CC	214	22	21	0.39 ± 0.15	0.38 ± 0.13	−0.03					
Hay River	HA	—	1	1	0.39 ± 0.49	—	—					
Maxhamish	MX	112	9	9	0.39 ± 0.19	0.37 ± 0.13	−0.05					
Snake Sahtaneh	SS	314	21	21	0.39 ± 0.15	0.38 ± 0.12	−0.03					
Westside Fort Nelson	WFN	203	7	7	0.39 ± 0.21	0.36 ± 0.14	−0.08					
East Williston	EW	—	3	3	0.39 ± 0.30	—	—	DU7	NM			
Finlay	FI	96	5	5	0.40 ± 0.24	0.36 ± 0.15	−0.11					
Frog	FR	206	7	6	0.39 ± 0.22	0.36 ± 0.14	−0.08					
Gataga	GA	179	6	5	0.39 ± 0.23	0.36 ± 0.15	−0.08					
Muskwa	MU	917	29	28	0.39 ± 0.14	0.39 ± 0.12	0.00					
Pink Mountain	PM	533	40	39	0.39 ± 0.13	0.39 ± 0.12	0.00					
A La Peche	ALP	150	18	14	0.39 ± 0.17	0.37 ± 0.13	−0.05	DU8	SM‐C		Southern cluster	CE
Kennedy Siding	KS	138	15	11	0.40 ± 0.18	0.37 ± 0.14	−0.08					
Klinse‐Za	KZ	138	37	25	0.39 ± 0.15	0.38 ± 0.13	−0.03					
Narraway	NA	35	3	3	0.40 ± 0.30	—	—					
Quintette	QI	140	20	17	0.39 ± 0.16	0.38 ± 0.13	−0.03					
Redrock‐Prairie Creek	RPC	96	11	10	0.40 ± 0.19	0.37 ± 0.14	−0.08					
Barkerville	BR	50	5	4	0.36 ± 0.26	0.31 ± 0.18	−0.16	DU9	SM‐S			
Hart Ranges	HR	510	66	59	0.39 ± 0.12	0.40 ± 0.11	0.03					
Narrow Lake	NL	8	2	2	0.33 ± 0.35	—	—					
North Cariboo	NC	169	24	23	0.39 ± 0.15	0.38 ± 0.12	−0.03					
Wells Gry South	WGS	187	19	18	0.37 ± 0.17	0.37 ± 0.14	0.00					
Groundhog	GR	46	6	3	0.36 ± 0.26	0.31 ± 0.18	−0.16					SE
Central Selkirks	CK	27	6	6	0.34 ± 0.24	0.31 ± 0.18	−0.10					
Columbia North	CN	219	49	33	0.36 ± 0.16	0.36 ± 0.15	0.00					
Columbia South	CS	4	2	2	0.32 ± 0.34	—	—					
Purcells South	PS	0	3	2	0.28 ± 0.32	—	—					
South Selkirks	SK	0	2	2	0.38 ± 0.36	—	—					
Banff	BNP	0	2	1	0.30 ± 0.40	—	—	DU8	SM‐C			J‐B
Brazeau	BZ	10	6	5	0.36 ± 0.25	0.31 ± 0.18	−0.16					
Maligne	ML	0	10	9	0.36 ± 0.20	0.35 ± 0.15	−0.03					
Tonquin	TQ	50	28	22	0.36 ± 0.17	0.35 ± 0.15	−0.03					

*Note:* Total number of genotypes remaining after quality filtering (*n* = 759) and excluding close relatives (Non‐relatives, *n* = 678) are provided for each subpopulation, along with observed heterozygosity (*H*_*o*_), expected heterozygosity (*H*_*e*_) and *F*_IS_ calculated while including close relatives. *H*_*e*_ and *F*_IS_ were only estimated for subpopulations containing at least 5 quality‐filtered samples. Each subpopulation's Designatable Unit (DU) and Species at Risk Act (SARA) classifications, as well as the major genetic cluster it was assigned to in hierarchical *Structure* analyses (down to level 3, as depicted on Figures 4 and 5), are also presented. SM‐N, SM‐C and SM‐S are abbreviations for Southern Mountain‐northern group, Southern Mountain‐central group and Southern Mountain‐southern group, respectively. II, NW, NE, CE, J‐B and SE are abbreviations for Itcha‐Ilgachuz, northwestern, northeastern, central‐eastern, Jasper‐Banff and southeastern, respectively. Population estimates (Pop est) are taken from the last available estimates (COSEWIC 2014; Government of Alberta 2017; Government of British Columbia 2023; Parks Canada 2018, 2024). Colours present in DU, SARA, and Structure 1–3 columns correspond to the DU, SARA grouping, or genetic cluster of each subpopulation.

### 2.3 Loci Mapping and Genotype Filtering

Positional data for the SNPs were originally provided for the ULRtarCaribou_2 genome scaffold‐level assembly (GCA_019903745.1) (Prunier et al. 2022). However, for our analysis we opted to remap these SNPs to the second version of this genome, ULRtarCaribou_2v2 genome (GCA_019903745.2), a chromosomal‐level assembly (Poisson et al. 2023). To do so, we aligned the source sequence data from the Illumina array manifest file (Appendix S2) to the ULRtarCaribou_2v2 genome assembly (Poisson et al. 2023). First we removed 13,445 uninformative or suboptimal loci as recommended by Carrier et al. (2022) (see Supporting Informations of Carrier et al. (2022) for the list of loci). Each SNP's source sequence was then formatted as an entry in a FASTA file and aligned to ULRtarCaribou_2v2 using bowtie2 v2.3.1 (Langmead and Salzberg 2012) and the *sensitive* alignment parameter. Samtools v1.6 (Li et al. 2009) was then used to call SNPs and obtain positions (see Appendix S1 for code and parameters).

Genotype filtering was performed as described in Michalak (2023). In brief, SNP genotypes received from the Centre d'Expertise et de Service Génome Québec were filtered using PLINK v1.9. First, we filtered for mapped SNPs (described above). Then a few samples identified as duplicates were removed using a > 95% similarity threshold and information from the PLINK *genome* function. The PLINK functions *list‐duplicate‐vars* and *exclude* were then used to identify and remove duplicate SNPs. Further filtering consisted of excluding individuals with < 95% genotyping rate (*mind* 0.05), as well as SNPs with < 95% genotyping rate (*geno* 0.05), deviating from Hardy–Weinberg equilibrium (*hwe* 1e‐6), with a minor allele frequency < 0.05 (*maf* 0.05) and in strong linkage disequilibrium with another SNP (*indep‐pairwise* 50 5 0.5). Additionally, we investigated the presence of loci under selection using F_ST_ outlier analysis which could bias estimates of neutral population structure (Holderegger et al. 2006); for this we used the R (R Core Team 2013) package *OutFLANK* v0.2 (Whitlock and Lotterhos 2015) and the function *pOutlierFinderChiSqNoCorr* using the default false discovery rate and a minimum per locus heterozygosity of 0.1. F_ST_ outlier analysis found zero loci to be under selection. Ultimately, 759 individuals from the 45 pre‐defined subpopulations (Table 1) and 32,440 SNPs were retained. A dataset excluding 81 close relatives (parent–child or full sibling relationships, where one individual from each pair was retained) was also generated for use in specific analyses. This was done with the same filtering pipeline following the exclusion of close relatives using the PLINK v2 (Chang et al. 2015) *king‐cutoff* command with a threshold of 0.177. This dataset contained a total of 678 individuals and 32,636 SNPs.

### 2.4 Genetic Diversity

To examine patterns of genetic diversity across the study area, we used the R package *dartR* v2.9.7 (Mijangos et al. 2022) to calculate observed heterozygosity (*H*_*o*_) of each pre‐defined subpopulation. We also calculated expected heterozygosity (*H*_*e*_) for each pre‐defined subpopulation with at least 5 genotyped individuals and inferred genetic clusters. We acknowledge that *n =* 5 is a low sample size for calculating heterozygosity, but this threshold was chosen to balance accuracy with the inclusion of subpopulations. These were then used to calculate *F*_IS_ based on the formula (*H*_e_−*H*_o_)/*H*_e_ (Wright 1949). We also estimated the degree of differentiation between pairs of pre‐defined subpopulations as well as inferred genetic clusters for which both expected and observed heterozygosity were available. This was accomplished using pairwise fixation index (*F*_ST_) values calculated using *StAMMP* v1.6.3 (Pembleton et al. 2013) in R, with significance assessed using 1000 bootstraps.

To assess if latitude (a proxy for position between the northern and southern glacial refugia) was associated with heterozygosity, we modelled *H*_*e*_ as a function of latitude and/or recent census size to account for the latter's likely relationship with heterozygosity. Population estimates were obtained from government reports (COSEWIC 2014; Government of Alberta 2017; Government of British Columbia 2023; Parks Canada 2018, 2024) and are reported in Table 1. The last population estimate for the Maligne subpopulation was 0 (as the subpopulation was extirpated); in this case a population estimate of 5 was used to allow for its inclusion in this analysis. Specifically, we examined a series of generalized linear models (GLM) with a Gaussian error distribution. In these models, we tested all combinations of census size and latitude fitted as different terms as fixed effects (Table S1). Census size was tested as either a linear or a log transformed term given that *H*_*e*_ should eventually plateau as census size increases. Latitude was tested as both a linear and quadratic term given that we expected higher subpopulation *H*_*e*_ values in the middle of our range where hybridisation has taken place. To select the best fitting model, we examined the *r* ^2^ values of the models and the Akaike Information Criterion corrected for small sample size (AIC_C_). These analyses were conducted in R using the *glm* function from the *stats* v4.2.1 package. Data visualization was performed using *ggplot2* v3.5.1 (Wickham 2011) and *Visreg* v2.7.0 (Breheny and Burchett 2017).

### 2.5 Population Genetic Structure

We assessed population genetic structure using a combination of model‐ and distance‐based approaches. For these analyses we excluded close relatives. As an initial assessment, we conducted a Principal Component Analysis (PCA) and a Discriminant Analysis of Principal Components (DAPC) using *adegenet* v2.1.10 (Jombart 2008; Jombart and Ahmed 2011) in R. The *find.clusters* function was used to examine all principal components (PCs) and identify the best‐fitting number of clusters given Bayesian Information Criterion (BIC) values for *K* clusters ranging from 1 to 45. We interpreted the best number of clusters as being the point where the curve of BIC values as a function of *K* elbowed (Thia 2023). To describe the clusters identified using DAPC we chose to retain all eigenvalues for *K*‐1 discriminant functions, as well as 44 PCs to match the number of pre‐defined subpopulations minus one, as recommended by Thia (2023). Finally, we incorporated the first two discriminant functions in a scatterplot to visualize variation among identified groups.

Population structure was further evaluated using the Bayesian clustering approach implemented in *Structure* v2.3.4 (Pritchard et al. 2000), which groups individual genotypes into *K* clusters that maximize within‐cluster Hardy–Weinberg and linkage equilibria. Because genetic structure likely occurs at multiple levels in woodland caribou we performed *Structure* analyses in a hierarchical fashion (see Vähä et al. (2007) for a description of this approach). We initially ran *Structure* ten times for each value of *K* from 1 to 10 using the admixture model, correlated allele frequencies and no a priori grouping of individuals. Each run consisted of a burn‐in of 20,000 iterations followed by 50,000 Markov chain Monte Carlo (MCMC) repetitions, which was assessed as adequate based on convergence. The *R* package *pophelper* v2.3.1 (Francis 2017) was then used to calculate the Δ*K* statistic of (Evanno et al. 2005) to examine which value of *K* was best supported by the data. Additionally, *pophelper* was used to consolidate clusters from multiple iterations of *Structure* and visualize results. As in Vähä et al. (2007), we then ran additional *Structure* analyses for each of the clusters identified using the same parameters as above. As the *ΔK* statistic cannot determine the presence of only one true genetic cluster (Janes et al. 2017), we visually examined Q‐matrices assignments to determine at which hierarchical level analyses should stop. Given our research objective of characterising broad‐scale genetic structure, we ceased analyses when clusters were being identified within pre‐defined subpopulations, as clusters below the subpopulation level likely represent family groups.

Population structure was also examined using the spatially explicit R program *TESS3* v1.0 (Caye et al. 2016) implemented in R. In contrast to *Structure*, *TESS* assigns individuals to clusters while incorporating information on each sample's geographic location. In instances where samples were not associated with a precise sampling location (*n =* 111), we assigned a sampling location in one of two ways. If precise sampling locations for other individuals from the same pre‐defined subpopulation was available, we assigned a random GPS location around the centroid of these known sampling locations within the maximum extent of the distribution of other subpopulation members. When precise locations were not known for any individual from the subpopulation, we generated random locations around a putative subpopulation central location, with the maximum extent of the distribution set as the average outer limit observed across all pre‐defined subpopulations with precise individual capture locations. For the *TESS3* analysis we conducted 10 runs for values of *K* ranging from 1 to 45 (tolerance = 1 × 10^−7^, max. iterations = 1000) and used the cross‐entropy criterion to select the optimal value of *K*, which corresponds to the one with the lowest cross‐validation score. We also used the *tess3r* v1.1.0 (Caye et al. 2016) R package to create maps of the geographic distribution of genetic clusters and their corresponding geographic boundaries.

We constructed an individual‐based neighbor‐joining tree using Manhattan distances from a genotype matrix calculated using the R package *BEDMatrix* v2.0.4 (Grueneberg and de los Campos 2019). The neighbor‐joining tree was inferred using the *nj* function in the *ape* v5.8 (Paradis et al. 2004; Paradis and Schliep 2019) R package and its confidence assessed using 1000 bootstraps. The tree was then visualized using the *ggtree* v3.15.0 R package (Yu et al. 2017).

### 2.6 Isolation‐By‐Distance

To examine how spatial separation influenced trends in genetic differentiation between subpopulations we tested for patterns of isolation‐by‐distance across the study area. We examined if Euclidean geographic distance between the centroid of capture locations for each subpopulation with at least five genotyped individuals was associated with Nei's genetic distance (Nei 1972, 1978) using a Mantel test with the R package *ade4* v1.7.22 (Chessel et al. 2004; Thioulouse et al. 1997). Since population structure can skew isolation‐by‐distance results when using Mantel tests (Meirmans 2012), we also performed separate tests for each major genetic cluster inferred at levels 2 and 3 of our hierarchical analysis.

### 2.7 Isolation‐By‐Landscape Features

To investigate the effect of specific landscape features on genetic differentiation, we used partial Mantel tests to assess whether features identified as boundaries in our clustering analysis influenced genetic distances between subpopulations, while controlling for geographic distance. We calculated Nei's genetic distances between subpopulations, geographic distances and constructed a binary matrix indicating whether subpopulation pairs were on the same (0) or opposite sides (1) of a given landscape feature. Only subpopulations with greater than five genotyped individuals were included in this analysis. All partial Mantel tests were conducted using the *vegan* package v2.7.1 in R.

## 3 Results

### 3.1 Genetic Diversity Within and Among Populations

**FIGURE 2:**
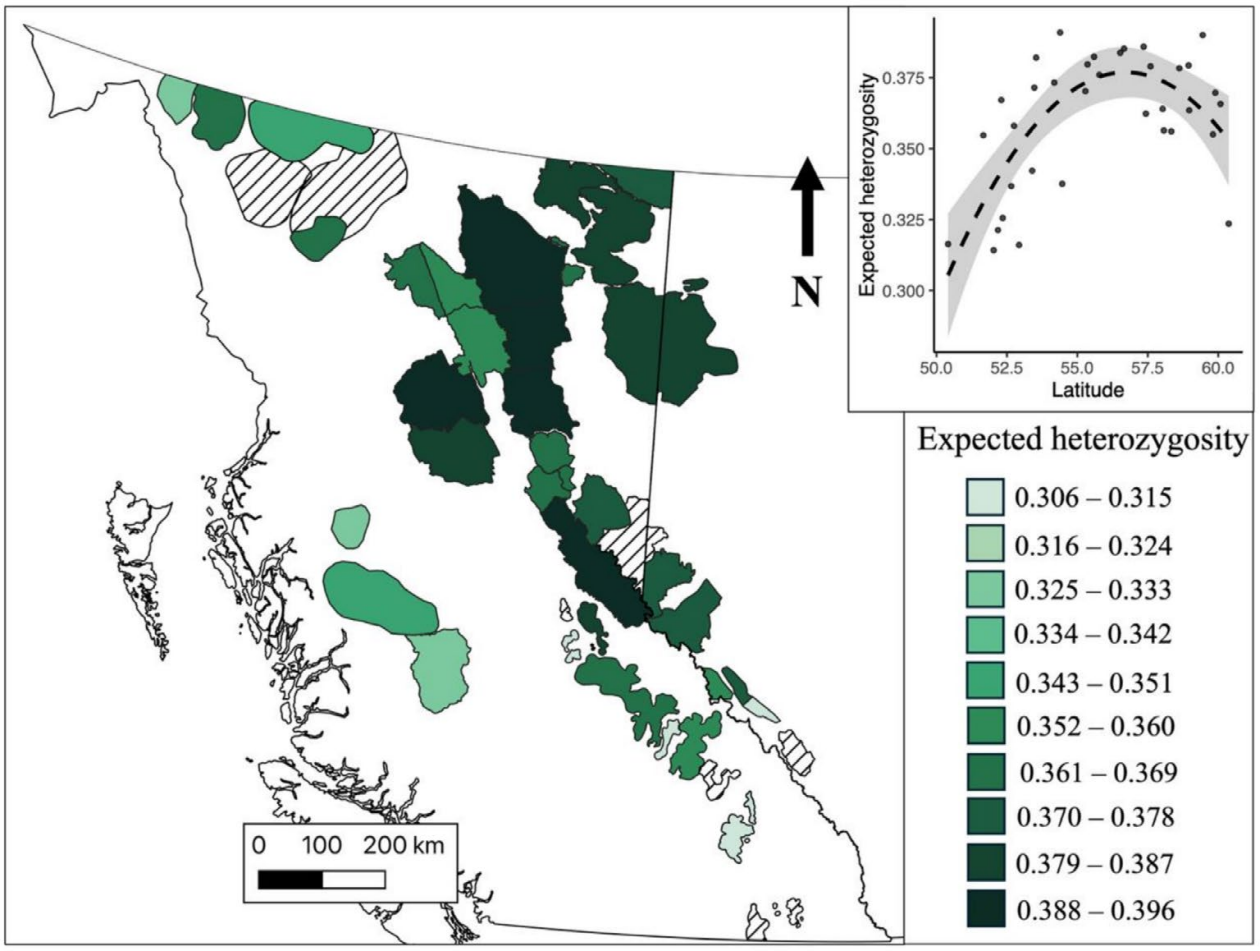
Expected heterozygosity across Woodland caribou subpopulations in western Canada (*n* = 45). Crosshatching represents subpopulations with less than five individuals genotyped, and outlines with no colour represent unsampled subpopulations. Insert in top right was generated using the R *visreg* function and shows the predicted association between latitude and expected heterozygosity from the best fitting linear model. The grey area depicts 95% confidence intervals.

Overall *H*_*o*_ and *H*_*e*_ were 0.38 ± 0.10 (s.d.) and 0.41 ± 0.10 and ranged from 0.28 to 0.40 and 0.31 to 0.40 within pre‐defined subpopulations, respectively (Table 1). Mean pairwise *F*_ST_ between all subpopulations was 0.08 (range −0.0005 to 0.18), with the greatest values observed between the Itcha‐Ilgachuz and Brazeau subpopulations, and the lowest between Finlay and Pink Mountain subpopulations (Appendix S3). *F*_IS_ ranged from −0.16 to 0.03 (Table 1). Notably, most *F*_IS_ values were negative, likely a result of gene flow between subpopulations given the low pairwise *F*_ST_ values observed across the sampling range. Genetic diversity varied across the sampling range, being highest in the centre and decreasing toward higher and lower latitudes. From our analysis of how latitude and population size affect *H*_*e*_, the best fitting model (model 8; *r* ^2^ = 0.56, AIC_C_ weight = 0.65) was supported over the next best model by a ΔAIC_C_ of 1.67 (Table S1). In the best fitting model, *H*_*e*_ increased with the linear term for latitude, decreased with the quadratic term for latitude and showed a near significant positive relationship with log‐transformed census size (Figure 2, Figure S1, Table S2).

### 3.2 Population Genetic Structure

**FIGURE 3:**
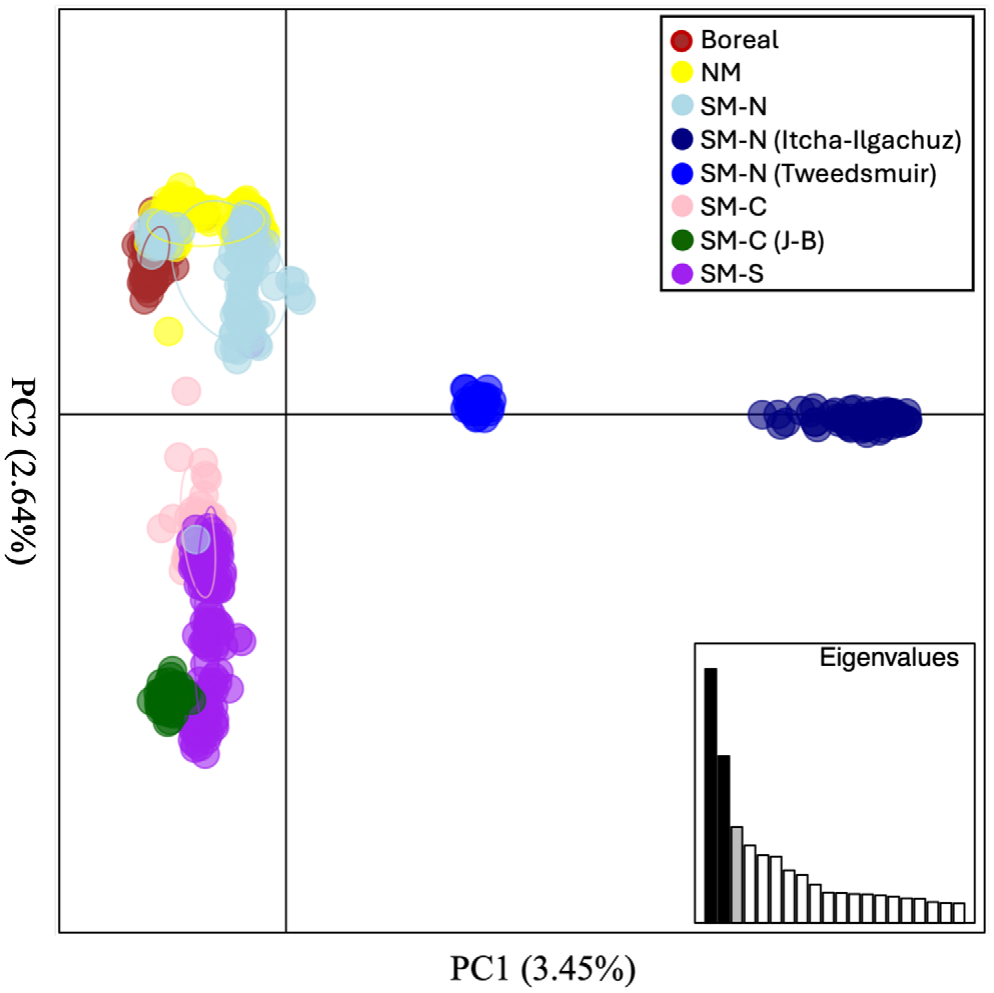
Principal Component Analysis (PCA) of woodland caribou in western Canada. Points in the PCA represent individuals, categorized according to their respective SARA groupings (SARA 2012a, 2012b, 2014). Itcha‐Ilgachuz and Tweedsmuir subpopulations, and Jasper‐Banff (J‐B) from the SM‐N and SM‐C groups, respectively, are highlighted to show discontinuities in the groupings. NM represents Northern Mountain.

A PCA of all individuals suggested the presence of multiple genetic clusters across the study range (Figure 3). The first PC, which explained 3.45% of the variation, mostly distinguished individuals belonging to the Itcha‐Ilgachuz and Tweedsmuir subpopulations of western British Columbia (BC) from all other subpopulations. The second PC, which explained 2.64% of the variation, primarily separated individuals found in the northern part of the sampled range from those found in the central and southern parts. The DAPC indicated the most optimal number of clusters to be between four and seven, and the resulting scatterplots exhibited patterns similar to the PCA (Figure S2).

**FIGURE 4:**
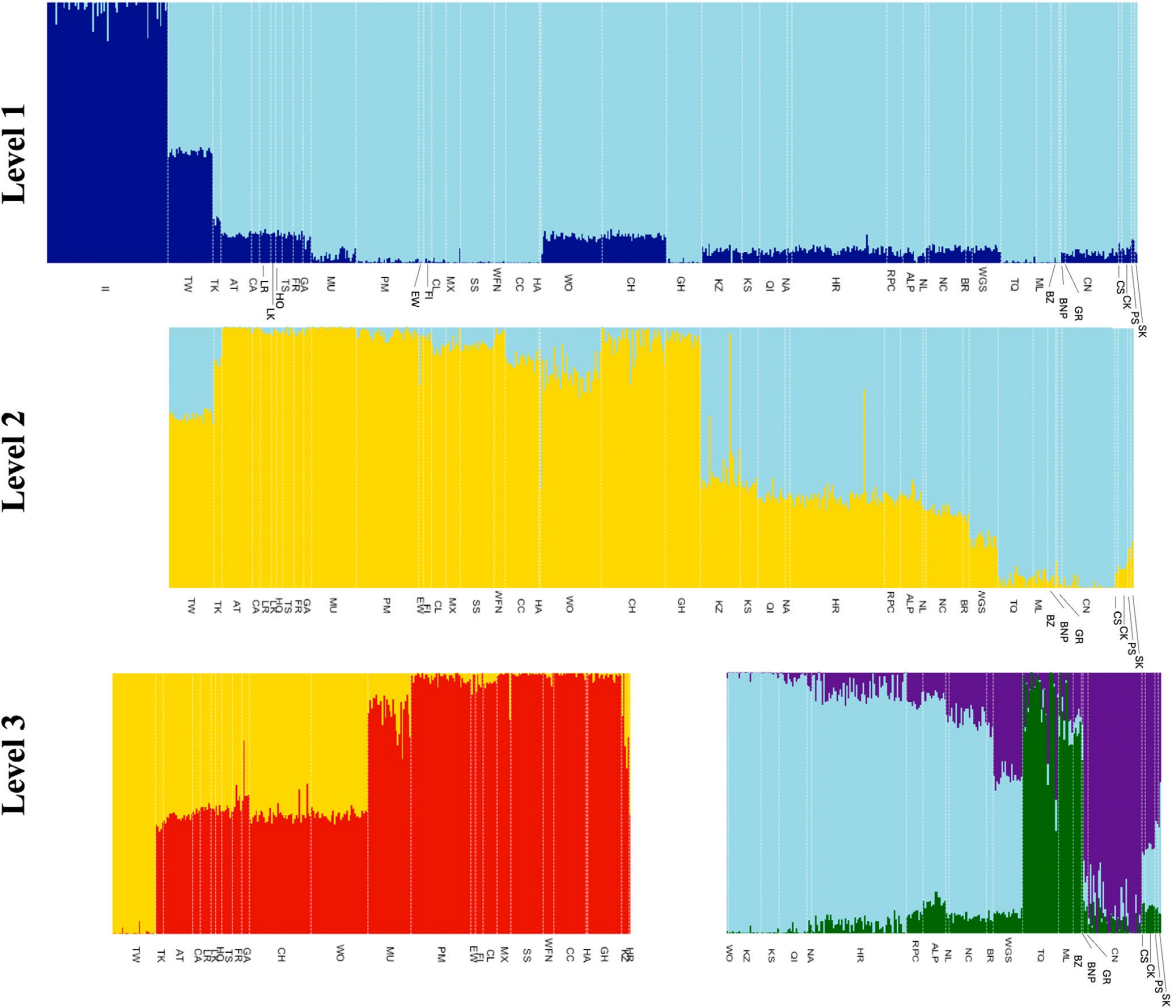
Admixture plots for all woodland caribou included in subpopulation structure analysis (*n =* 678), at level 1 all individuals were included in the analysis, at level 2 all individuals except Itcha‐Ilgachuz individuals were included in the analysis (dark blue at level 1), at level 3 analysis was run on the two separate clusters identified at level 2. Labels correspond to subpopulations, for full information on subpopulations see Table 1.

**FIGURE 5:**
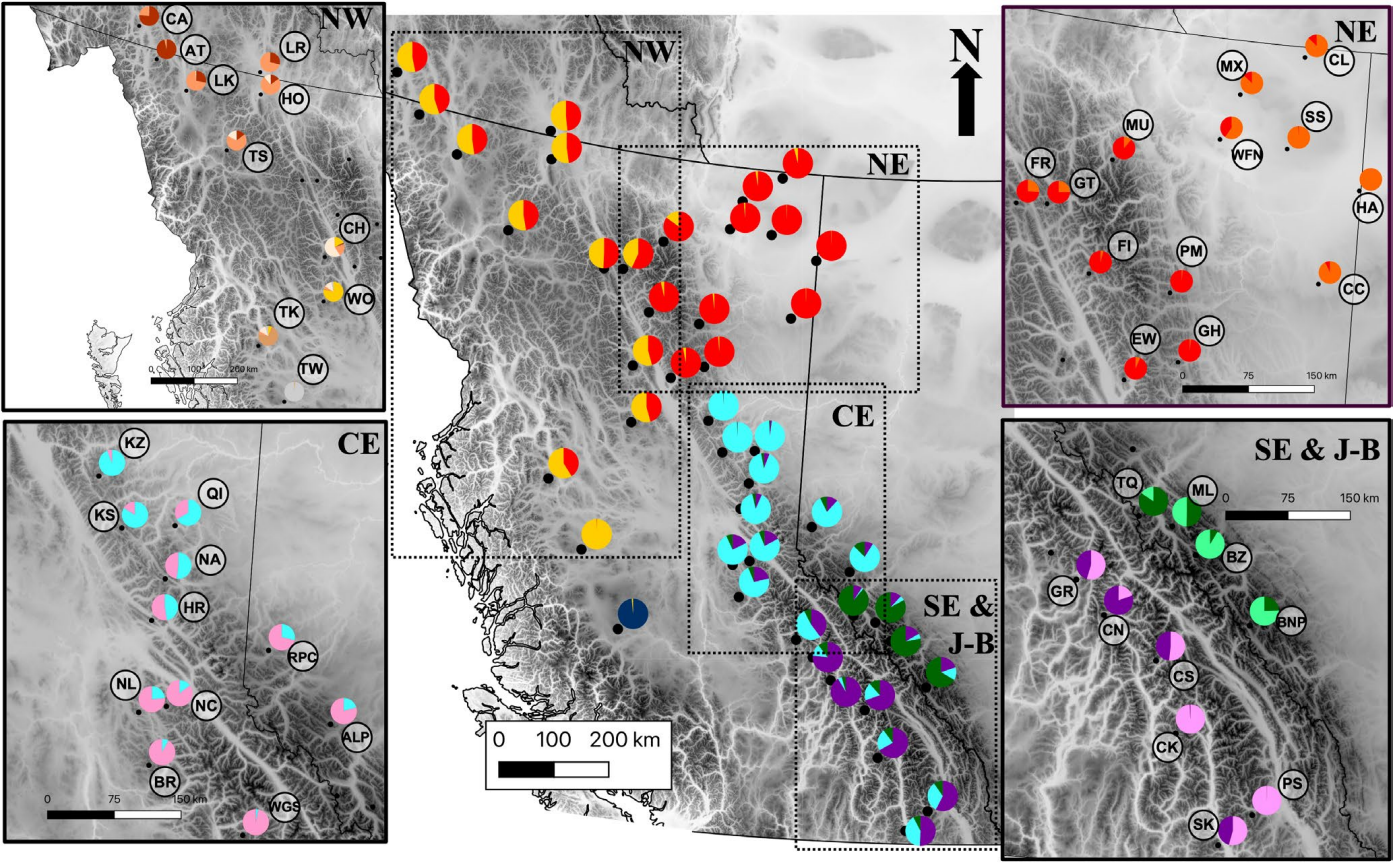
Woodland caribou subpopulations in western Canada and their admixture of each of the genetic clusters identified in *Structure* analysis at different hierarchical levels. Main panel shows the six main genetic clusters identified in western Canada, top left shows genetic clusters identified within the northwestern cluster (NW), top right shows genetic clusters identified within the northeastern cluster (NE), bottom left shows genetic clusters identified within the central‐eastern cluster (CE), and bottom right shows genetic clusters identified within the Jasper‐Banff (J‐B) local population unit cluster and southeastern cluster (SE and J‐B). Dashed lines represent landscape features which define cluster boundaries; Rocky Mountain Trench (black), Peace River (blue), Yellowhead Pass and Athabasca River (red), North Thompson Valley (pink), Great Divide (yellow). For subpopulation names, major clusters and other details see Table 1.

The hierarchical *Structure* analysis resulted in six levels of clustering. The Δ*K* plot of the initial analysis including all samples indicated *K =* 2 as the most supported number of clusters (Figure S3), with the Itcha‐Ilgachuz subpopulation clustering separately from all other subpopulations (Figure 4 [level 1]). The subsequent analysis of all remaining samples (excluding Itcha‐Ilgachuz) also indicated *K =* 2 as the most supported number of clusters within this reduced group of samples (Figure S4), which indicated a northern cluster and a southern cluster (Figure 4 [level 2], Table 1). The *ΔK* plots for the resulting northern (Figure S5) and southern (Figure S6) clusters further supported dividing the northern cluster in two; resulting in a northeastern and northwestern, and the southern cluster into three; resulting in a central‐eastern, a Jasper‐Banff and a southeastern cluster (Table 1, Figure 4 [level 3], Figure 5). Ultimately, hierarchical clustering analyses beyond the aforementioned six major clusters (Figures 4 and 5) found the 45 predefined subpopulations to separate out into a total of 31 clusters (Table S3, Figures S7–S21). These finer‐resolution clusters generally consisted of a single pre‐defined subpopulation, but in some cases pre‐defined subpopulations were grouped together (e.g., Klinse‐Za and Kennedy Siding) (Figures S7–S21, Table S3).

In the *TESS* entropy criterion plot, cross‐validation values continuously declined up to *K* = 45 (the largest *K* tested; Figure S22). *TESS'*s geographic predictions for values of *K* = 2–8 are presented in Figure S22. These broadly align with the clusters identified by *Structure*, except in the *TESS* analysis the Tweedsmuir subpopulation separates as its own cluster from *K* = 6 prior to separation of the Jasper‐Banff cluster at *K = 7*.

**FIGURE 6:**
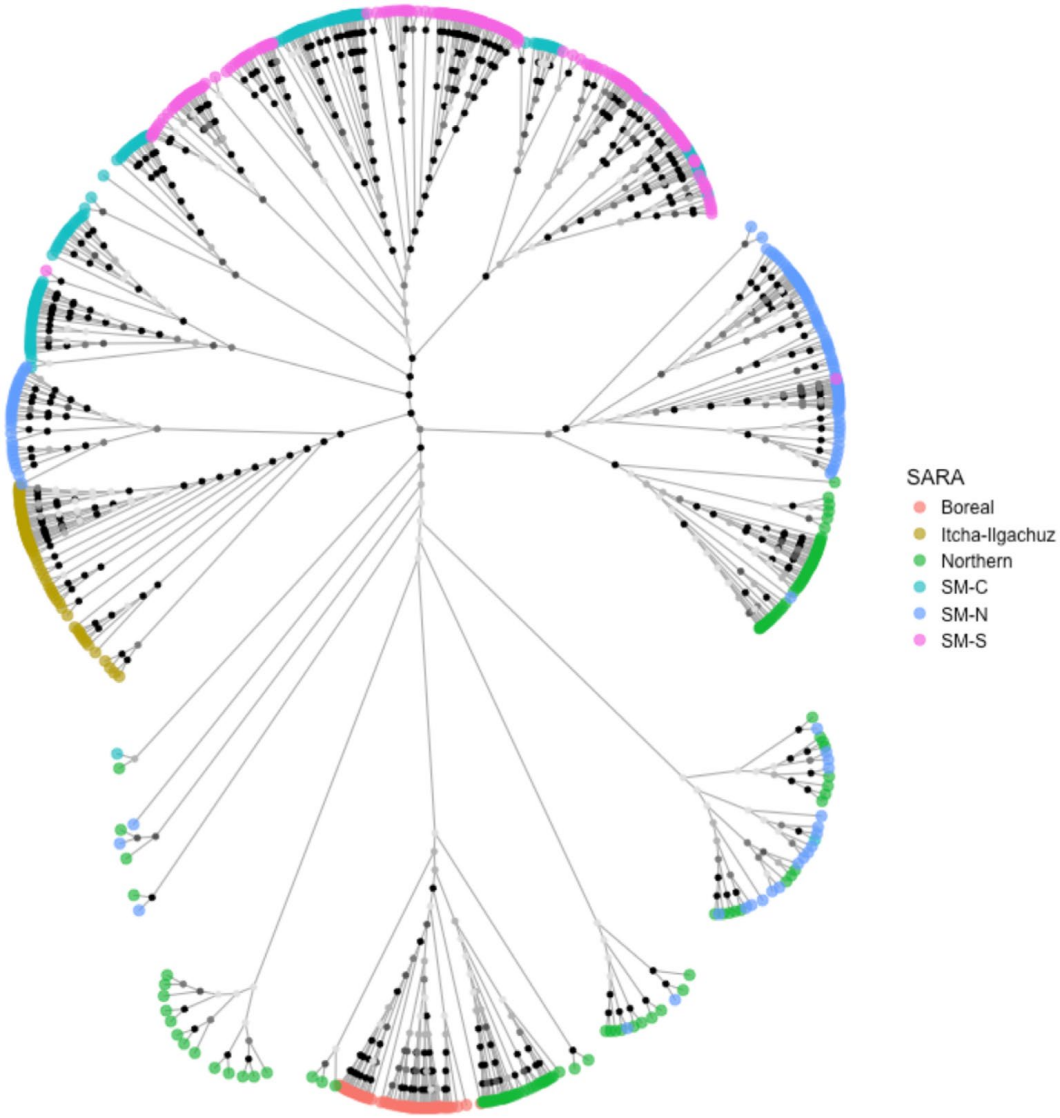
Neighbor‐joining tree of woodland caribou sampled throughout western Canada. Branches represent individuals, with tip colours representing each individual's SARA listing (SARA 2014) (For a more detailed figure which includes source subpopulation see Figure S23). Bootstrap values were estimated based on 1000 replicates and are represented on internal nodes as circles in five classes/shades of grey (in 20% increments, with the darkest circle representing the 81%–100% class). SM‐N, Southern Mountain‐northern group; SM‐C, Southern Mountain‐central group; SM‐S, Southern Mountain‐southern group. Note that the SM‐N branch beside Itcha‐Ilgachuz is composed of all Tweedsmuir individuals.

The neighbor‐joining tree closely mirrored population structure results from other analyses, showing a clear distinction between Itcha‐Ilgachuz, Southern Mountain‐northern group (SM‐N), Northern Mountain and Boreal. Southern Mountain‐central (SM‐C) group and Southern Mountain‐southern (SM‐S) group were separate from all other aforementioned groups but were intermixed with each other. Within these groups, individuals clustered by subpopulation but not by SARA subgroups or COSEWIC DUs. Itcha‐Ilgachuz and Tweedsmuir individuals (SM‐N) formed a distinct branch, separate from all other Northern Mountain, SM‐N and Boreal subpopulations. The remaining SM‐N and Northern Mountain individuals were intermixed and appeared on the same branch as all Boreal individuals (which clustered together) (Figure 6, Figure S23).

### 3.3 Genetic Variation Within and Among Major Clusters

*H*_*e*_ and *H*_*o*_ within and *F*_ST_ between major clusters were calculated post hoc after identification during the *Structure* analysis. At the upper hierarchical structure levels (1–3), *H*_*e*_ and *H*_*o*_ ranged from 0.33 to 0.41 and 0.33 to 0.38, respectively. Itcha‐Ilgachuz had the lowest *H*_*e*_ and *H*_*o*_, whereas the level 1 main cluster had the highest *H*_*e*_ and the northeastern cluster at level 3 had the highest *H*_*o*_ (Table 2). Across levels 1–3, *F*_ST_ values ranged from 0.02 to 0.15 and were all significantly different from zero after Bonferroni correction (Table 3). The lowest and highest levels of differentiation were observed at level 3, the lowest between the northeastern and northwestern clusters (*F*_ST_ = 0.02) and the highest between the Itcha‐Ilgachuz and the Jasper‐Banff clusters at level 3 (*F*_ST_ = 0.15), with the mean *F*_ST_ for level 3 being 0.07. Pairwise *F*_*ST*_ values between clusters at levels 4 to 7 ranged from < 0.001 to 0.342 (Appendix S3), while *H*_*e*_ and *H*_*o*_ ranged from 0.19–0.40 and 0.29–0.40 within these clusters (Appendix S4).

**TABLE 2:** Observed (*H*_*o*_) heterozygosity, expected (*H*_*e*_) heterozygosity values and associated standard deviations, and *F*_*IS*_ for inferred clusters at level 4–7 of our hierarchical analysis of woodland caribou in western Canada. Cluster labels follow the abbreviations in Table 1 and levels presented in Figure 4.

Level/cluster	*H*_*o*_	*H*_*e*_	*F*_IS_
Level 1			
Itcha‐Ilgachuz	0.329 ± 0.173	0.327 ± 0.166	< 0.001
All other subpopulations	0.383 ± 0.095	0.409 ± 0.099	0.063
Level 2			
Itcha‐Ilgachuz	0.329 ± 0.173	0.327 ± 0.166	< 0.001
Northern Cluster	0.387 ± 0.099	0.407 ± 0.101	0.051
Southern Cluster	0.378 ± 0.106	0.399 ± 0.108	0.053
Level 3			
Itcha‐Ilgachuz	0.329 ± 0.173	0.327 ± 0.166	< 0.001
NW	0.380 ± 0.112	0.398 ± 0.111	0.048
NE	0.393 ± 0.105	0.405 ± 0.103	0.033
CE	0.389 ± 0.110	0.400 ± 0.108	0.030
SE	0.352 ± 0.146	0.369 ± 0.140	0.044
J‐B	0.359 ± 0.157	0.360 ± 0.142	0.017

**TABLE 3:** Pairwise *F*_ST_ values between inferred genetic clusters for woodland caribou in western Canada.

Level/cluster		
Level 1	Itcha‐Ilgachuz	All other subpopulations
Itcha‐Ilgachuz	—	< 0.001
All other subpopulations	0.083 (0.082–0.084)	—

Level 2	Itcha‐Ilgachuz	Northern Cluster	Southern Cluster
Itcha‐Ilgachuz	—	< 0.001	< 0.001
Northern Cluster	0.086 (0.085–0.087)	—	< 0.001
Southern Cluster	0.095 (0.094–0.096)	0.024 (0.024–0.024)	—

Level 3	Itcha‐Ilgachuz	NW	NE	CE	SE	J‐B
Itcha‐Ilgachuz	—	< 0.001	< 0.001	< 0.001	< 0.001	< 0.001
NW	0.086 (0.085–0.087)	—	< 0.001	< 0.001	< 0.001	< 0.001
NE	0.101 (0.100–0.103)	0.023 (0.023–0.024)	—	< 0.001	< 0.001	< 0.001
CE	0.132 (0.130–0.134)	0.059 (0.058–0.060)	0.062 (0.061–0.063)	—	< 0.001	< 0.001
SE	0.096 (0.094–0.097)	0.030 (0.029–0.030)	0.028 (0.028–0.029)	0.038 (0.037–0.038)	—	< 0.001
J‐B	0.149 (0.147–0.151)	0.072 (0.071–0.074)	0.070 (0.069–0.071)	0.051 (0.050–0.052)	0.067 (0.066–0.068)	—

*Note:* *F*_ST_ values and 95% confidence intervals are given below the diagonal, and respective *p*‐values above the diagonal. Cluster labels follow abbreviations defined in Table 1 and presented in Figure 4.

### 3.4 Isolation‐By‐Distance

**FIGURE 7:**
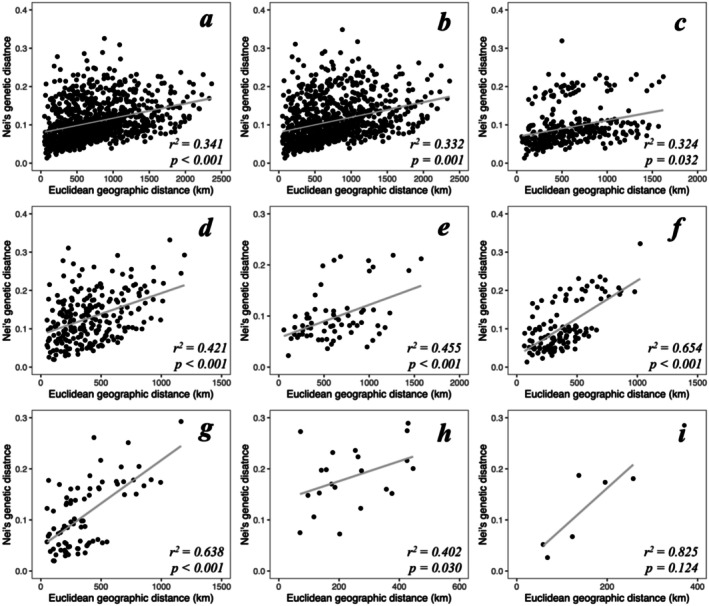
Nei's genetic distances as a function of Euclidean geographic distance (km) between predefined subpopulations of woodland caribou in western Canada. Subpopulations were only included in the analysis if they had greater than five individuals genotyped. Panels depict analyses including (a) all subpopulations included in the study, or limited to those from (b) the main cluster at level 1, (c) the northern and (d) southern cluster at level 2, or the (e) northwestern, (f) northeastern, (g) central‐eastern, (h) Jasper‐Banff and (i) southeastern clusters at level 3.

Geographic distance explained 34.1% of the variation in Nei's genetic distance across the sampled range (Mantel test, *p* < 0.001, Figure 7a). When testing for isolation‐by‐distance within each cluster at each hierarchical level up to level 3 (except the Itcha‐Ilgachuz cluster which could not be tested as it only contained a single subpopulation), we identified significant or near‐significant patterns of isolation‐by‐distance in all clusters (Figure 7).

### 3.5 Isolation by Landscape Features

When examining the geographic distribution of major genetic clusters, numerous landscape features appeared to be associated with the boundaries between genetic clusters. The Peace River and its drainage appeared to separate the northern and southern clusters at level 2; the northern extent of the Rocky Mountain Trench appeared to separate the northeastern and northwestern clusters; a combination of the North Thompson River and the headwaters of the Fraser River separate the Central‐eastern and southeastern clusters; a combination of the Yellowhead Pass and Athabasca River separate the Central‐eastern and Jasper‐Banff clusters; and the Great Divide (the high elevation mountains along the border of Alberta and BC) appeared to separate the southeastern and Jasper‐Banff clusters (Figures 1, 4 and 5 [level 2]). Post hoc analyses indicated that four out of these five landscape features likely affected population structure. The Peace River and its drainage explained 5.9% of the variation in Nei's genetic distance between subpopulations located north and south of the river in the main range (excluding Itcha‐Ilgachuz; *p* < 0.001). North of the Peace River, separation across the Rocky Mountain Trench accounted for 16.3% of the variation in Nei's genetic distance between the northeastern and northwestern subpopulations (*p* < 0.001). South of the Peace River, separation across the North Thompson River and headwaters of the Fraser River explained 41.9% of the variation in Nei's genetic distance (*p* = 0.032), separation across the Yellowhead Pass and Athabasca River explained 43.0% of the variation in Nei's genetic distance (*p* = 0.032) and South of the North Thompson River and Athabasca River, 41.5% of the variation in Nei's genetic distance was attributed to separation east and west of the Great Divide; however, this result was only near‐significant (*p* = 0.10).

## 4 Discussion

We built on the preliminary work of Michalak (2023) to conduct a comprehensive hierarchical analysis of genetic diversity and variation in woodland caribou across western Canada. We found varying levels of genetic differentiation between subpopulations of woodland caribou in western Canada, explained by a combination of post‐glacial recolonisation patterns, isolation‐by‐distance and landscape features. Our clustering analysis identified hierarchical population structure ranging from *K =* 2 at its highest level to *K =* 31 at the lowest level, with *K =* 6 appearing to be an appropriate broad scale grouping for capturing unique genetic diversity. Patterns of genetic diversity across the landscape showed a tendency for higher diversity in the middle of the range, likely a result of secondary contact and historical hybridisation following post‐glacial recolonisation. This study presents a comprehensive assessment of woodland caribou population structure in western Canada, which provides essential information which can be considered in the conservation of woodland caribou in the region.

### 4.1 Broad‐Scale Spatial Genetic Structure

Woodland caribou in western Canada were found to exhibit a multi‐level hierarchical population genetic structure, consisting of two to six broad‐scale genetic clusters. The greatest degree of separation was found between the Itcha‐Ilgachuz subpopulation and all other subpopulations. We subsequently found an overarching north–south split among most subpopulations (all subpopulations when excluding Itcha‐Ilgachuz) at the Peace River and its drainage which we attribute to a combination of glacial history in this region (Shafer et al. 2010) and separation by river system. Beyond this, six clusters broadly represent the Itcha‐Ilgachuz subpopulation, northwestern BC, northeastern BC, central BC and Alberta, southeastern BC and the Jasper‐Banff region. This more localised population structure also appeared to result from limited gene flow across geographical features, particularly lowland habitats.

As previously mentioned, the most prominent segregation in the observed hierarchical population genetic structure is the separation of Itcha‐Ilgachuz and all other subpopulations, which indicates Itcha‐Ilgachuz possesses unique genetic variation which potentially results from either genetic drift or adaptation. In addition to the genetic differences reported here and elsewhere (Serrouya et al. 2012; Taylor et al. 2020), this subpopulation exhibits differing behavioural tendencies (Hughes et al. 2025; Lamb et al. 2025) when compared to other subpopulations in western Canada. However, the underlying mechanisms responsible for the unique genetic signature remain unclear. Notably, Itcha‐Ilgachuz caribou were distinct from both the northern and southern clusters at level 2, representative of the BEL and NAL ancestries. When comparing *F*_*ST*_ distances between Itcha‐Ilgachuz and other clusters, Itcha‐Ilgachuz appears to be most similar to the northwestern cluster, likely explained by its geographic proximity to other subpopulations within this cluster. However, contrary to this, previous studies have identified the subpopulation to be most similar and share ancestry with subpopulations in central and southeastern BC (Taylor et al. 2021, 2024). One explanation for the separation of Itcha‐Ilgachuz is that the subpopulation has been historically isolated (Taylor et al. 2020), and genetic drift has led to substantial genetic differentiation from other subpopulations. While further research will be needed to clarify this, our results suggest that Itcha‐Ilgachuz is on its own evolutionary trajectory, likely with its own unique adaptive potential and hence should be managed and conserved as such.

In the main range of the subpopulations (all subpopulations other than Itcha‐Ilgachuz) we identified a north–south split at the Peace River, representing the two glacial ancestries known to be present in this region. We observed a gradual rather than an abrupt shift from one ancestry to the other, and a low *F*_*ST*_ distance between the two clusters, indicative of a hybrid zone between the two lineages. This finding aligns with studies in other species (Shafer et al. 2010) and other studies of woodland caribou in this region (Cavedon, Poissant, et al. 2022; Cavedon, vonHoldt, et al. 2022; Serrouya et al. 2012; Taylor et al. 2021; Yannic et al. 2014). Furthermore, this north–south divide is also reflected in some behavioural differences (Apps et al. 2001; Hughes et al. 2025; Lamb et al. 2025; Theoret et al. 2022). Interestingly, individuals from the boreal region, thought to stem from the NAL (Cavedon, Poissant, et al. 2022; Cavedon, vonHoldt, et al. 2022), grouped within the northern (BEL ancestry) cluster. This suggests that contemporary gene flow between boreal and more northern subpopulations to their west may have weakened historical differentiation between these groups.

Major genetic clusters appeared to be delineated by prominent features of the landscape. We found major lowland habitat features to delineate population structure between some clusters in our study range, similar to other alpine adapted species (Deakin et al. 2020; Fedy et al. 2008; Sim et al. 2019). Two sub‐clusters were identified within the main northern cluster: a northeastern and northwestern cluster. These clusters were defined by, and likely are a result of, reduced gene flow across the northern Rocky Mountain Trench. However, extremely low *F*_ST_ values observed between these clusters suggest long‐range movements of caribou in this region (Watters and DeMars 2017) may lessen the effect of the barrier. We found the boundary between the central‐eastern cluster and the southeastern cluster delineated by the North Thompson River and headwaters of the Fraser River west of The Great Divide. East of the Great Divide, the central‐eastern cluster and Jasper‐Banff clusters are delineated by the Yellowhead Pass and Athabasca River. Conversely, high elevation habitats along the Great Divide appeared to potentially separate the southeastern and Jasper‐Banff clusters, similar to other species where high alpine and mountainous habitats limit dispersal (Ghaedi et al. 2021; Machado et al. 2018; Rueness et al. 2003; Zalewski et al. 2009). However, this result was only near significant when tested with a partial Mantel test, likely due to our limited sample sizes in this analysis and region. It should be recognised that despite the apparent large effects of these features on genetic distance between major clusters, up to ~43% in some cases, the total genetic distance between clusters at this level and among subpopulations in general tended to be low. These results further highlight how semi‐permeable features of the landscape can influence gene flow and in turn population genetic structure, whether these features are energetically expensive habitats to traverse (Olah et al. 2017; Pérez‐Espona et al. 2008) or patches of undesirable habitat such as areas of high predation risk (Deakin et al. 2020).

### 4.2 Fine‐Scale Spatial Structure

Spatial genetic structure was characterised by a pattern of isolation‐by‐distance, where geographic distance explained approximately a third of genetic distance between all pairs of subpopulations, likely resulting from both historical isolation and contemporary gene flow. The pattern of isolation‐by‐distance across the study area is lower than observed in other habitat‐specialised ungulates in this region, such as bighorn sheep ( *Ovis canadensis canadensis* ) (Deakin et al. 2020; Forbes and Hogg 1999) and mountain goats ( *Oreamnos americanus* ) (Shafer et al. 2011), likely because woodland caribou are highly mobile and occupy highly fragmented habitats (Maltman et al. 2024), which may weaken the signal of isolation‐by‐distance.

Ultimately, our hierarchical population structure analysis identified many sub‐clusters. These appeared to result from multiple causes including landscape features, geographic distance and behaviour. For example, in the northeastern cluster we observed a split between Northern Mountain and Boreal individuals (SARA 2012a, 2012b). Other breaks at lower hierarchical levels may be due to other landscape features, which are difficult to detect due to the numerous valleys, waterways and habitats present in this highly heterogenous landscape. Overall, we found a total of 31 subpopulation level clusters, suggesting that not all the 45 pre‐defined subpopulations are genetically distinct. This indicates that in some cases multiple predefined subpopulations may function as one or have become geographically isolated relatively recently.

### 4.3 Patterns of Genetic Diversity

We observed patterns of genetic diversity concordant with the history of post‐glacial recolonisation of the area (Hewitt 2004; Shafer et al. 2010, 2011). Typically, it is expected that genetic diversity should decrease with distance from source populations due to the founder effect (Frankham 1997), but here we found genetic diversity to be lower in the northwest and southeast and highest in the middle of the sampled range. The elevated genetic diversity in the middle of our sampling range is likely due to hybridisation following secondary contact between the two historical lineages in this region (Barton and Hewitt 1985; Canestrelli et al. 2010; Cavedon, Poissant, et al. 2022; McDevitt et al. 2009). Although subpopulations in the central part of this range are among some of the most at risk (Lamb et al. 2024), their elevated genetic diversity means they may also possess more adaptive potential and thus be more resilient to future environmental and habitat changes.

### 4.4 Conservation Implications

As suggested by the preliminary analyses of Michalak (2023), boundaries between major genetic clusters do not align with currently recognised units in the study region. While four of the six major genetic clusters identified somewhat resemble existing DU and SARA classification schemes (COSEWIC 2011; SARA 2012a, 2012b, 2014), boundaries between genetic clusters differ and additional genetic clusters appear to delineate variation in the region (Figure 1). To better capture genetic diversity and variance, the boundary between the Boreal and the Northern Mountain DU and SARA units could be shifted westward to the Northern Rocky Mountain Trench to include northern subpopulations east of this lowland system, forming northeastern and northwestern clusters. In the southern DU and SARA units, boundary redefinition could also be considered to reflect the differences between the central‐eastern, southeastern and newly identified Jasper‐Banff clusters; with a north–south split delineated by the North Thompson River, Fraser River headwaters and Athabasca River, and an east–west split south of this along the Great Divide (separating out the Jasper‐Banff region). Additionally, our analyses revealed that the Itcha‐Ilgachuz subpopulation forms a unique genetic cluster, highly distinct from all others (Figure 1).

All broad‐scale genetic clusters (levels 1–3) exhibit significant genetic differentiation. However, as highlighted by Hoelzel (2023), estimates of genetic differentiation with vast numbers of markers may overstate the significance of these differences. While gene flow between major clusters appears reduced, genetic differences should be considered in the context of overall variation, especially when factoring in behavioural distinctions (COSEWIC 2011; SARA 2012a, 2012b, 2014; Theoret et al. 2022) and known differences in glacial ancestry (McDevitt et al. 2009; Taylor et al. 2021; Yannic et al. 2014).

## 5 Conclusion

We built on the preliminary work of Michalak (2023) to characterise genetic diversity across woodland caribou range in western Canada. We identified a hierarchical population structure composed of multiple populations and subpopulations, which is best described by six genetic clusters: the northeastern, northwestern, central‐eastern, southeastern, the Jasper‐Banff and Itcha‐Ilgachuz clusters. This structure is indicative of post‐glacial recolonisation, particularly a north–south split, with patterns of hybridisation shaping genetic diversity across the landscape. This study exemplifies how wide‐ranging, mobile species can exhibit intricate population genetic structure, especially those with complex natural histories occupying highly heterogeneous landscapes.

## Funding

This research was financially supported by the Government of British Columbia, the Canadian Wildlife Service, Parks Canada and the Natural Sciences and Engineering Research Council of Canada Grant (ALLRP/561434–2020) to JP and MM. MM was funded by the European Union—NextGenerationEU, under the National Recovery and Resilience Plan (NRRP), Project title ‘National Biodiversity Future Center ‐NBFC’ (CN_00000033). AM was supported by an Alberta Graduate Excellence Scholarship.

## Conflicts of Interest

The authors declare no conflicts of interest.

## Supporting information


**Figure S1:** Predicted associations between (a) latitude (degrees North) and (b) log‐transformed census size and expected heterozygosity of woodland caribou subpopulations in western Canada from a linear mixed model. Plots generated using the R *visreg* function. Points show changes in response while holding all other variables constant. Grey area depicts 95% confidence intervals of predicted associations.
**Figure S2:** Scatterplot of Discriminant Analysis of Principal Components (DAPC) for woodland caribou ( *Rangifer tarandus caribou* ) in western Canada. When retaining 44 principal components and indicating a separation of individuals into four (a), five (b), six (c) and seven (d) clusters.
**Figure S3:** Log likelihood plots generated by pophelper for level 1 of Bayesian analysis of genetic clustering patterns of woodland caribou ( *Rangifer tarandus caribou* ) in western Canada.
**Figure S4:** Log likelihood plots generated by pophelper for level 2 of Bayesian analysis of genetic clustering patterns of woodland caribou ( *Rangifer tarandus caribou* ) in western Canada.
**Figure S5:** Log likelihood plots generated by pophelper for northern cluster level 3 of Bayesian analysis of genetic clustering patterns of woodland caribou ( *Rangifer tarandus caribou* ) in western Canada.
**Figure S6:** Log likelihood plots generated by pophelper for southern cluster level 3 of Bayesian analysis of genetic clustering patterns of woodland caribou ( *Rangifer tarandus caribou* ) in western Canada.
**Figure S7:** Admixture plots for woodland caribou ( *Rangifer tarandus caribou* ) included in subpopulation structure analysis from the northwestern cluster (*n =* 337). Labels correspond to individuals' putative subpopulations. Dashed boxes indicate what we consider to be resolved subpopulations.
**Figure S8:**. Log likelihood plots generated by pophelper for northeastern cluster level 4 of Bayesian analysis of genetic clustering patterns of woodland caribou ( *Rangifer tarandus caribou* ) in western Canada.
**Figure S9:**. Log likelihood plots generated by pophelper for northwestern cluster level 4 of Bayesian analysis of genetic clustering patterns of woodland caribou ( *Rangifer tarandus caribou* ) in western Canada.
**Figure S10:**. Log likelihood plots generated by pophelper for Boreal cluster level 5 of Bayesian analysis of genetic clustering patterns of woodland caribou ( *Rangifer tarandus caribou* ) in western Canada.
**Figure S11:**. Log likelihood plots generated by pophelper for northeastern slopes cluster level 5 of Bayesian analysis of genetic clustering patterns of woodland caribou ( *Rangifer tarandus caribou* ) in western Canada.
**Figure S12:**. Log likelihood plots generated by pophelper for Atlin and Carcross cluster level 5 of Bayesian analysis of genetic clustering patterns of woodland caribou ( *Rangifer tarandus caribou* ) in western Canada.
**Figure S13:**. Log likelihood plots generated by pophelper for Horseranch, Tsenaglode, Level‐Kawdy, Little Rancheria, Frog, Wolverine and Chase cluster level 5 of Bayesian analysis of genetic clustering patterns of woodland caribou ( *Rangifer tarandus caribou* ) in western Canada.
**Figure S14:**. Log likelihood plots generated by pophelper for the northern Boreal cluster level 6 of Bayesian analysis of genetic clustering patterns of woodland caribou ( *Rangifer tarandus caribou* ) in western Canada.
**Figure S15:** Admixture plots for woodland caribou ( *Rangifer tarandus caribou* ) included in subpopulation structure analysis from the central‐eastern cluster (*n =* 266). Labels correspond to individuals' putative subpopulations.
**Figure S16:**. Log likelihood plots generated by pophelper for central‐eastern cluster at level 4 of Bayesian analysis of genetic clustering patterns of woodland caribou ( *Rangifer tarandus caribou* ) in western Canada.
**Figure S17:**. Log likelihood plots generated by pophelper for southeastern cluster at level 4 of Bayesian analysis of genetic clustering patterns of woodland caribou ( *Rangifer tarandus caribou* ) in western Canada.
**Figure S18:**. Log likelihood plots generated by pophelper for northern Jasper‐Banff cluster at level 4 of Bayesian analysis of genetic clustering patterns of woodland caribou ( *Rangifer tarandus caribou* ) in western Canada.
**Figure S19:**. Log likelihood plots generated by pophelper for northern central‐eastern cluster at level 4 of Bayesian analysis of genetic clustering patterns of woodland caribou ( *Rangifer tarandus caribou* ) in western Canada.
**Figure S20:**. Log likelihood plots generated by pophelper for the southern central‐eastern cluster at level 5 of Bayesian analysis of genetic clustering patterns of woodland caribou ( *Rangifer tarandus caribou* ) in western Canada.
**Figure S21:**. Log likelihood plots generated by pophelper for the southeastern cluster at level 5 of Bayesian analysis of genetic clustering patterns of woodland caribou ( *Rangifer tarandus caribou* ) in western Canada.
**Figure S22:** *TESS* cross validation scores and predictions of genetic and geographic clusters inferred from *TESS K* = 2–8 for 678 woodland caribou ( *Rangifer tarandus caribou* ) distributed across 45 pre‐defined subpopulations in western Canada. Maps created using the *tess3r* package in R.
**Figure S23:** Neighbor‐joining tree of woodland caribou ( *Rangifer tarandus caribou* ) *sampled* throughout British Columbia and part of Alberta. Branches represent individuals, with tip colours representing each individual's source subpopulation and SARA listing (SARA 2014). Bootstrap values were estimated based on 1000 replicates and are represented on internal nodes as circles in five classes/shades of grey (in 20% increments, with the darkest circle representing the 81%–100% class). SM‐N, Southern Mountain‐Northern Group; SM‐C, Southern Mountain‐Central Group; SM‐S, Southern Mountain‐Southern Group.
**Table S1:** Models fitted to investigate the association of census size and latitude with expected heterozygosity. df, degrees freedom; AIC_C_, Akaike Information Criterion corrected for small sample size; ΔAIC_C_, delta AIC_C_.
**Table S2:** Parameter estimates for the best fitting model describing expected heterozygosity in woodland caribou subpopulations in western Canada.
**Table S3:** 31 final subpopulation level clusters identified by Bayesian clustering analysis of woodland caribou subpopulations in western Canada. Numbers in each cell denotes the number of individuals from that putative subpopulation in each cluster.

**Appendix S2:** The Illumina manifest file for the SNP assay used for genotyping all individuals in the study.

**Appendix S3:** Pairwise *F*_ST_ values between clusters identified by hierarchical admixture analysis at levels 4–7.

**Appendix S4:** Expected heterozygosity, observed heterozygosity and *F*_IS_ values for clusters identified by hierarchical admixture analysis at levels 4–7.

## Data Availability

Genetic data is provided on Dryad. Due to the sensitive nature of the species release of precise individual geospatial data is forbidden. Access to geospatial data may be granted following completion of data sharing agreements with the appropriate government agencies.
